# Sitagliptin decreases ventricular arrhythmias by attenuated glucose-dependent insulinotropic polypeptide (GIP)-dependent resistin signalling in infarcted rats

**DOI:** 10.1042/BSR20150139

**Published:** 2016-03-16

**Authors:** Tsung-Ming Lee, Wei-Ting Chen, Nen-Chung Chang

**Affiliations:** * Department of Medicine, Cardiology Section, China Medical University-An Nan Hospital, Tainan 70911, Taiwan; † Department of Medicine, China Medical University, Taichung 40447, Taiwan; ‡ Cardiovascular Research Laboratory, China Medical University Hospital, Taichung 40447, Taiwan; § Division of Cardiology, Department of Internal Medicine, School of Medicine, College of Medicine, Taipei Medical University, Taipei 11031, Taiwan; ∥ Division of Cardiology, Department of Internal Medicine, Taipei Medical University Hospital, Taipei 11031, Taiwan

**Keywords:** glucose-dependent insulinotropic polypeptide, myocardial infarction, nerve growth factor, resistin, sympathetic nerve

## Abstract

Myocardial infarction (MI) was associated with insulin resistance, in which resistin acts as a critical mediator. We aimed to determine whether sitagliptin, a dipeptidyl peptidase-4 (DPP-4) inhibitor, can attenuate arrhythmias by regulating resistin-dependent nerve growth factor (NGF) expression in postinfarcted rats. Normoglycaemic male Wistar rats after ligating coronary artery were randomized to either vehicle or sitagliptin for 4 weeks starting 24 h after operation. Post-infarction was associated with increased myocardial noradrenaline [norepinephrine (NE)] levels and sympathetic hyperinnervation. Compared with vehicle, sympathetic innervation was blunted after administering sitagliptin, as assessed by immunofluorescent analysis of tyrosine hydroxylase, growth-associated factor 43 and neurofilament and western blotting and real-time quantitative RT-PCR of NGF. Arrhythmic scores in the sitagliptin-treated infarcted rats were significantly lower than those in vehicle. Furthermore, sitagliptin was associated with reduced resistin expression and increased Akt activity. *Ex vivo* studies showed that glucose-dependent insulinotropic polypeptide (GIP) infusion, but not glucagon-like peptide-1 (GLP-1), produced similar reduction in resistin levels to sitagliptin in postinfarcted rats. Furthermore, the attenuated effects of sitagliptin on NGF levels can be reversed by wortmannin (a phosphatidylinositol 3-kinase antagonist) and exogenous resistin infusion. Sitagliptin protects ventricular arrhythmias by attenuating sympathetic innervation in the non-diabetic infarcted rats. Sitagliptin attenuated resistin expression via the GIP-dependent pathway, which inhibited sympathetic innervation through a signalling pathway involving phosphatidylinositol 3-kinase (PI3K) and Akt protein.

## Abstract (precis)

The rats after myocardial infarction (MI) exposed to sitagliptin, a dipeptidyl peptidase-4 inhibitor, led to attenuated sympathetic innervation probably through a phosphatidylinositol 3-kinase and Akt-dependent pathway.

## INTRODUCTION

Dipeptidyl peptidase-4 (DPP-4) inhibitors are a novel class of drugs used to treat hyperglycaemia. DPP-4 inhibitors act by inhibiting the degradation of glucagon-like peptide-1 (GLP-1) and glucose-dependent insulinotropic polypeptide (GIP) via the plasma DPP-4 enzyme [1]. The widespread expression of DPP-4 in tissues including myocardium suggests that this protein may play a role in cardiovascular function. DPP-4 inhibitors have been shown to provide cardioprotection in a glucose-independent manner via pleiotropic effects [1]. This cardioprotective effect of DPP-4 inhibitors has also been observed in normoglycaemic rats [2]. Resistin is a newly identified adipokine that has been suggested to play a role in the development of insulin resistance [3]. Administration of recombinant resistin in rodents has been shown to impair insulin sensitivity [4,5], and treatment with anti-resistin antisense oligonucleotides has been shown to reverse insulin resistance [6]. Increasing evidence indicates that resistin plays an important regulatory role in addition to its role in insulin resistance in cardiovascular disease. Resistin overexpression has been associated with impaired myocardial remodelling and cardiac dysfunction in rats [7]. In addition, although resistin mRNA expression levels have been reported to be the highest in white adipose tissue in rodents, resistin has also been found to be expressed in the heart [8]. Resistin has also been shown to impair glucose transport in isolated cardiomyocytes [9] and to be up-regulated by cyclic stretch and aorta-caval shunt [10], suggesting that resistin mRNA is expressed in cardiomyocytes.

During the chronic stage of myocardial infarction (MI), regional increases in sympathetic innervation have frequently been observed at the remote zone [11]. In addition, increased sympathetic nerve density has been shown to be responsible for the occurrence of lethal arrhythmias and sudden cardiac death in humans [12]. Nerve growth factor (NGF) belongs to the neurotrophin family of proteins, which play an essential role in the differentiation, survival and synaptic activity of peripheral sympathetic and sensory nervous systems [13]. A number of cell types have been shown to secrete NGF, including fibroblasts [14], cardiac myocytes [15] and vascular endothelial cells [16].

The effect of DPP-4 inhibitors on resistin is controversial. The chronic use of DPP-4 inhibitors has been associated with reduced levels of resistin in mice [17], however sitagliptin cannot suppress resistin levels in patients with type 2 diabetes mellitus [18]. Resistin has been shown to exert variable responses on sympathetic nerve activity in different tissues. Sympathetic drive to the renal tissues has been reported to be increased after centrally administered resistin by activating phosphatidylinositol 3-kinase (PI3K), however sympathetic tone has been shown to be decreased in brown adipose tissue [19]. There are therefore clearly cell type-specific responses to resistin. Very recently, we have demonstrated that PI3K pathway is a signalling pathway involving NGF expression and post-infarction sympathetic innervation [20]. The effect of resistin on myocardial sympathetic activity remains unclear, and whether sitagliptin can attenuate NGF expression by directly regulating the expression and secretion of resistin is unknown. The aim of the present study was to investigate whether sitagliptin regulates resistin expression, and to investigate the molecular mechanism involved and the possible functional relevance of this regulation on arrhythmia in a rat MI model.

## METHODS

### Animals

All of the rats received humane care, and the experiment was approved and conducted in accordance with local institutional guidelines of China Medical University for the care and use of laboratory animals and conformed with the Guide for the Care and Use of Laboratory Animals published by the US National Institutes of Health (NIH Publication No. 85-23, revised 1996).

#### Experiment 1 (*in vivo*)

Healthy non-diabetic male Wistar rats (300–350 g) were subjected to ligation of the left anterior descending artery as previously described [21], resulting in infarction of the left ventricular (LV) free wall. The rats were randomly assigned into either vehicle (saline) or sitagliptin (5 mg/kg per day, Merck) groups, administered orally by gastric gavage once a day. This dose of sitagliptin has been shown to maintain blood glucose levels at the same level as in vehicle-treated rats, so that we could directly assess the effect of the drugs independently of blood glucose control [22]. Treatment was initiated 24 h after infarction when they would be most beneficial [23]. The study was designed to last for 4 weeks because myocardial remodelling in rats (70–80%) is mostly complete within 3 weeks [24]. Sham-operated rats served as controls to exclude the possibility that the drug itself directly altered sympathetic innervation. In each treated group, treatment was stopped ∼24 h before the end of the experiments in order to eliminate any pharmacological action.

#### Experiment 2 (*ex vivo*)

To examine the relative importance of GIP and GLP-1 in sitagliptin-related resistin levels, we used infusions of GIP and GLP-1, respectively, in an *ex vivo* model. Four weeks after the induction of MI by coronary ligation, the infarcted rat hearts were isolated and subjected to no treatment (vehicle), sitagliptin (5 μM), GIP (100 nM), GLP-1 (100 nM), or the combination of sitagliptin and GIP. The doses of sitagliptin, GIP and GLP-1 have been shown to be effective in modulating biological activities [25,26]. Noncirculating modified Tyrode's solution was used to perfuse each heart, containing glucose 5.5 mM, NaCl 117.0 mM, NaHCO_3_ 23.0 mM, KCl 4.6 mM, NaH_2_PO_4_ 0.8 mM, MgCl_2_ 1.0 mM and CaCl_2_ 2.0 mM, equilibrated at 37°C with a 95% O_2_ and 5% CO_2_ gas mixture. Given that resistin secretion was evident within 1 h following GIP treatment [26], the drugs were infused for 60 min. All of the hearts (*n*=5 each group) were used for western blot analysis for resistin protein at the remote zone (>2 mm outside the infarct).

#### Experiment 3 (*ex vivo*)

To assess the role of the PI3K/Akt pathway in sitagliptin-induced NGF suppression, infarcted rat hearts were treated with the PI3K specific inhibitor, wortmannin (WM), together with sitagliptin. MI was induced by coronary ligation, and 4 weeks later the infarcted rat hearts were isolated and subjected to no treatment (vehicle), sitagliptin (5 μM), sitagliptin + WM (100 nM, Sigma–Aldrich) or sitagliptin + resistin (10 nM). The drugs were perfused for 60 min. The doses of WM [27] and resistin [27] used in that study have been used previously. All of the hearts (*n*=5 each group) were used for western blot analysis for NGF at the remote zone.

### Haemodynamics and infarct size measurements

Haemodynamic parameters and infarct size were measured in anesthetized rats at the end of the study as described in detail in the Supplementary material online.

### *Ex vivo* electrophysiological studies

As central sympathetic activity may confound the effect of ventricular arrhythmias induced by pacing, we used the Langendorff heart technique. For a detailed method, please refer to the Supplementary material online.

### Real-time RT-PCR of resistin and NGF

mRNAs were quantified by real-time RT-PCR with *cyclophilin* as a loading control. For a detailed method, please refer to the Supplementary material online.

### Western blot analysis of resistin, Akt and NGF

Samples obtained from the remote zone at week 4 after infarction. The primary antibodies were resistin (Chemicon), p-Akt1 (ser473, Cell Signaling Technology), Akt1 (Santa Cruz Biotechnology), NGF (Chemicon) and β-actin (Santa Cruz Biotechnology). For a detailed method, please refer to the Supplementary material online.

### Immunofluorescent studies of tyrosine hydroxylase, growth-associated factor 43 and neurofilaments

In order to investigate the spatial distribution and quantification of sympathetic nerve fibres, analysis of immunofluorescent staining was performed on LV muscle from the remote zone. The analysis of the immunofluorescent staining is described in detail in the Supplementary material online.

### Laboratory measurements

We measured the activity of DPP-4 and levels of active GIP and GLP-1 in plasma to confirm that the administration of sitagliptin was associated with the suppression of plasma DPP-4 activity and increase in active GIP levels. Given that GIP, but not GLP-1, modulates resistin levels [26], we also measured GIP levels in the present study. EDTA plasma was used to measure the total levels of GIP (Millipore Corporation), GLP-1 (Millipore Corporation) and DPP-4 activity (Quantizume Assay System, BIOMOL International). Insulin was measured using an ultrasensitive rat enzyme immunoassay (Mercodia).

Even though cardiac innervation was detected by immunofluorescent staining of tyrosine hydroxylase, growth-associated factor 43, and neurofilaments, this did not necessarily mean that the nerves were functional. Therefore, to examine sympathetic nerve function after the administration of sitagliptin, we measured levels of LV noradrenaline [norepinephrine (NE)] in the samples obtained from the remote zone. Myocardium was minced and suspended in 0.4 N perchloric acid with 5 mmol/L reduced GSH (pH 7.4), homogenized with a polytron homogenizer for 60 s in 10 vol. The total level of NE was measured using a commercial ELISA kit (Noradrenalin ELISA, IBL Immuno-Biological Laboratories).

### Statistical analysis

The results are presented as mean ± S.D. All statistical analyses were performed using SPSS software (SPSS, version 12.0, Chicago, Illinois). Differences between groups were tested by ANOVA. When there was a significant effect, between group differences were compared using Bonferroni's correction. Electrophysiological data (programmed electrical stimulation-induced arrhythmia score) were compared using the Kruskal–Wallis test followed by the Mann–Whitney test. A *P* value less than 0.05 was considered to indicate statistical significance.

## RESULTS

### Part 1. *In vivo* study (Experiment 1)

**Figure 1:**
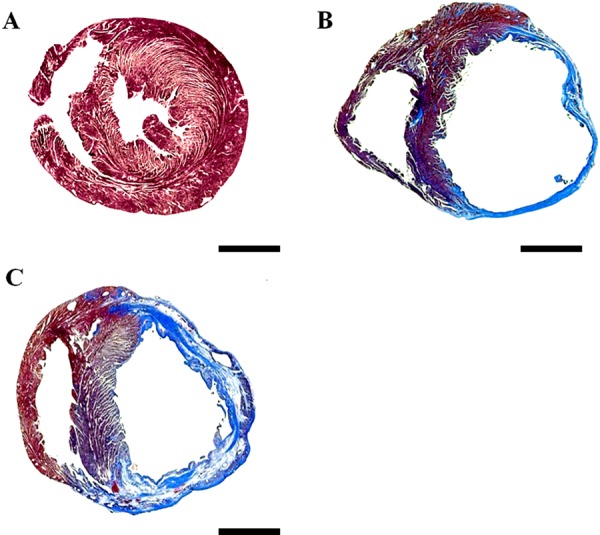
Effect of sitagliptin-treated hearts on infarct size 4 weeks after MI stained by Masson's trichrome **(A**) Sham; (**B**) infarction treated with vehicle; (**C**) infarction treated with sitagliptin. Bar=2 mm.

There were no differences in mortality between the two infarcted groups throughout the study. Sitagliptin had little effect on the gross morphology of the hearts in the sham-operated group. Four weeks post-infarction, the infarcted areas of the left ventricle were very thin and had been totally replaced by fully differentiated scar tissue (Figure 1). The weight of the left ventricle inclusive of the septum between the infarcted groups remained basically the same for all 4 weeks (Table 1). Compared with the vehicle-treated group, the maximum rates of LV +d*P*/d*t* and -d*P*/d*t* were significantly increased, and the lung weight (LungW)/body weight (BW) ratio was significantly lower in the sitagliptin-treated group, consistent with favourable LV remodelling. There were no differences in LV end-systolic pressure and infarct size between the infarcted groups, and there were no changes in plasma glucose or insulin level between the two groups.

**Table 1:** Cardiac morphology, haemodynamics and plasma glucose, GLP-1, GIP, DPP-4, insulin and tissue NE levels at the end of study Values are mean ± S.D. LVEDP, left ventricular end-diastolic pressure; LVESP, left ventricular end-systolic pressure; LVW, left ventricular weight; NE, norepinephrine levels from remote myocardium; RVW, right ventricular weight. *P<0.05 compared with vehicle-treated sham; ^†^*P*<0.05 compared with vehicle-treated infarcted group.

	Sham		Infarction treated with	
Parameters	Vehicle	Sitagliptin	Vehicle	Sitagliptin
No. of rats	12	10	11	11
Body weight, g	365±8	378±2	376±10	385±11
Heart rate, bpm	411±10	402±8	408±12	405±12
LVESP, mm Hg	103±7	101±6	95±5	97±6
LVEDP, mm Hg	4±2	4±2	19±4*	16±4*
+d*P*/d*t*, mm Hg/s	8234±342	7982±285	2672±318*	3389±329*†
-d*P*/d*t*, mm Hg/s	6982±278	6763±259	2382±306*	3072±303*†
Infarct size, %	–	–	40±2	41±2
LVW/BW, mg/g	2.54±0.20	2.83±0.18	3.98±0.35*	3.48±0.41*
RVW/BW, mg/g	0.59±0.14	0.62±0.15	1.38±0.19*	1.22±0.18*
LungW/BW, mg/g	4.34±0.35	4.18±0.42	6.99±0.38*	5.14±0.47†
Glucose, mg/dl	90±6	91±5	93±4	87±7
GLP-1, pmol/l	6.5±0.4	16.8±1.8*	7.1±0.7	18.6±1.4*†
GIP, pg/ml	109±21	188±28*	126±51	215±34*†
DPP-4 activity	1.56±0.15	0.42±0.19*	1.67±0.12	0.39±0.16*†
Insulin, μu/ml	18±12	21±16	67±18*	72±12*
LV NE, μg/g protein	1.01±0.25	1.16±0.17	2.85±0.21*	1.29±0.43†

### Plasma GLP-1, GIP, DPP-4 activity and NE levels

Levels of plasma GLP-1, GIP, and NE and DPP-4 activity were measured to confirm that the oral delivery of sitagliptin had been successful (Table 1). The results showed a significant increase in the levels of GLP-1 and GIP in the sitagliptin-treated group, and a 77% reduction in plasma DPP-4 activity in the sitagliptin-treated group compared with the vehicle group.

We then determined LV NE levels to determine the possible role of cardiac NE synthesis. The results showed that the administration of sitagliptin did not affect the concentration of NE in the tissue of the sham-operated rats (data not shown). However, the LV NE levels were significantly up-regulated by 2.82-fold in the vehicle-treated rats compared with the sham-operated group (2.85±0.21 compared with 1.01±0.25 μg/g protein, *P*<0.001, Table 1). Sitagliptin administration significantly reduced LV NE levels compared with the vehicle group.

### Immunofluorescent analyses

**Figure 2:**
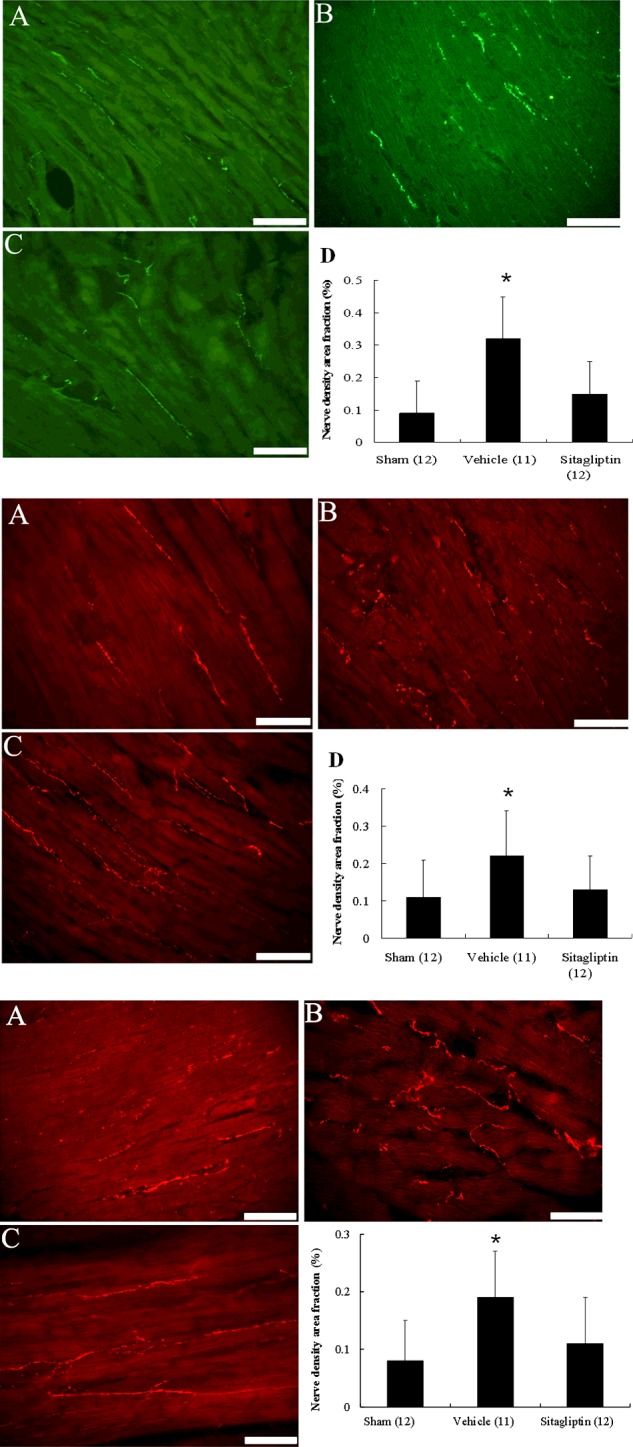
Upper, immunofluorescent staining for tyrosine hydroxylase from the remote regions (magnification 400×) Tyrosine hydroxylase-positive nerve fibres are located between myofibrils and are oriented longitudinal direction as that of the myofibrils. **Middle**, immunofluorescent staining for growth-associated protein 43 from the remote regions (magnification 400×). **Lower**, immunofluorescent staining for neurofilament from the remote regions (magnification 400×). (**A**) Sham; (**B**) infarction treated with vehicle; (**C**) infarction treated with sitagliptin. Bar=50 μm. nerve density area fraction (%) at the remote zone. Each column and bar represents mean ± S.D. *P<0.05, compared with sham and sitagliptin.

In immunofluorescent analysis, the nerve fibres immunostained with tyrosine hydroxylase were oriented in the longitudinal axis of adjacent myofibres (Figure 2, upper panel). In addition, the tyrosine hydroxylase-positive nerve density was significantly increased in the vehicle group compared with the sham group. The sitagliptin-treated rats had a lower nerve density at the remote regions compared with the vehicle-treated rats (0.32±0.13% compared with 0.09±0.10% in the sitagliptin group, *P*<0.05). Similarly, the densities of growth-associated protein 43 and neurofilament-positive (Figure 2, middle and lower panels) nerves were significantly attenuated in the sitagliptin-treated group compared with the vehicle-treated group. These morphometric results mirrored the NE findings.

### Resistin, Akt and NGF protein

**Figure 3:**
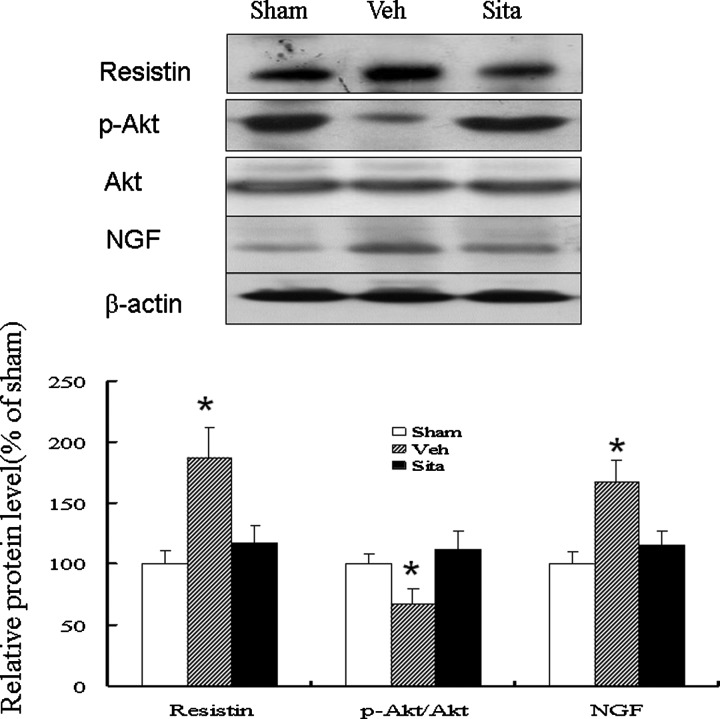
Western blot analysis of resistin, Akt and NGF (MW: 13 kDa) in homogenates of the LV from the remote zone When compared with vehicle-treated infarcted rats, sitagliptin-treated infarcted rats had significantly higher p-Akt levels and lower resistin and NGF levels at the remote zone by quantitative analysis. Relative abundance was obtained by normalizing the protein density against that of β-actin. Results are mean ± S.D. of three independent experiments. *P<0.05, compared with sham and sitagliptin.

Western blot analysis of resistin, Akt and NGF protein showed that the resistin levels were significantly up-regulated by 1.87-fold at the remote zone in the vehicle group compared with the sham group (*P*<0.001, Figure 3). Compared with the vehicle group, the sitagliptin group had a significantly lower resistin level at the remote zone. In addition, sitagliptin treatment resulted in a significant increase (*P*<0.01) in the relative p-Akt (ser473) level (112±15%) compared with vehicle treatment (67±13%). Treatment with sitagliptin also significantly decreased the NGF level by 31% compared with the vehicle group.

### Resistin and NGF mRNA expression

**Figure 4:**
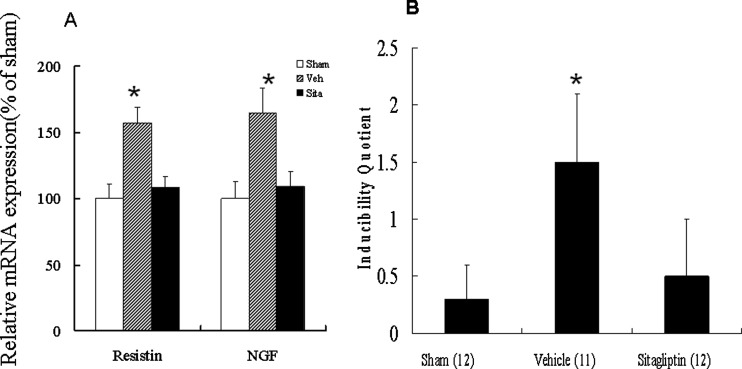
mRNA expression and ventricular arrhythmias (**A**) Left ventricular resistin and NGF mRNA expression. Each mRNA was corrected for an mRNA level of cyclophilin. Each column and bar represents mean ± S.D. *P<0.05, compared with sham and sitagliptin. (**B**) Inducibility quotient of ventricular arrhythmias by programmed electrical stimulation 4 weeks after MI in an *ex vivo* model. *P<0.05, compared with sham and sitagliptin.

PCR amplification of the cDNA showed that the expression of *resistin* mRNA was up-regulated by 1.57-fold at the remote zone in the vehicle group compared with the sham group (*P*<0.001, Figure 4A). However, the expression of *resistin* mRNA was significantly decreased in the sitagliptin group compared with the vehicle group. The expression of *NGF* mRNA changed in parallel with the expression of *resistin* mRNA.

### Electrophysiological stimulation

We then investigate the physiological effect of attenuated sympathetic hyperinnervation using ventricular pacing. The arrhythmia score in the sham group was very low (0.3±0.3) (Figure 4B), whereas ventricular tachyarrhythmias consisting of ventricular tachycardia and ventricular fibrillation were induced by stimulation in the vehicle group. Sitagliptin treatment significantly decreased the inducibility of ventricular tachyarrhythmias compared with the vehicle group.

### Part 2. *Ex vivo* study

#### Effect of GIP and GLP-1 on sitagliptin-induced resistin levels (Experiment 2)

**Figure 5:**
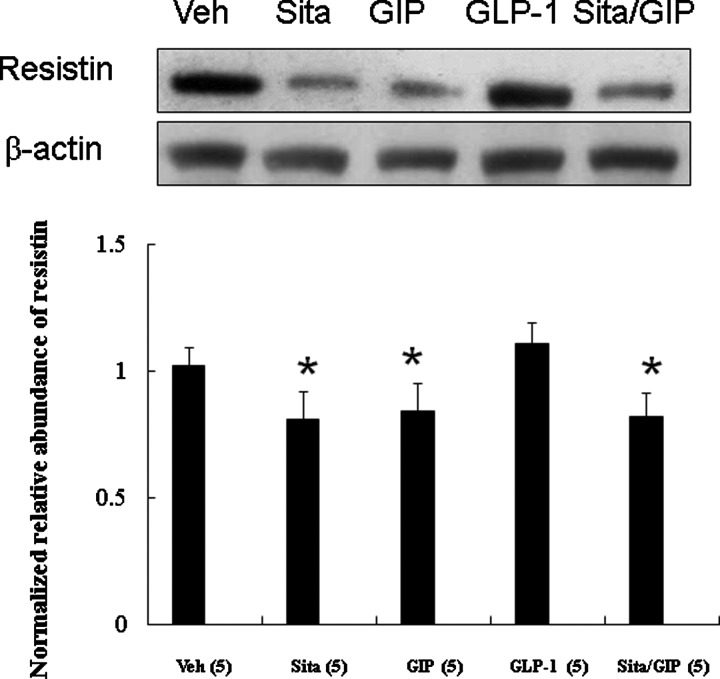
Effect of GIP and GLP-1 on sitagliptin-induced resistin levels (Experiment 2) In a rat isolated heart model, effect of GIP and GLP-1 on resistin levels. Compared with sitagliptin-treated infarcted rats, resistin levels were significantly higher in rats infused with GLP-1. Relative abundance was obtained by normalizing the density of resistin protein against that of β-actin. Each point is an average of three separate experiments (*n*=5 per group). *P<0.05 compared with the groups treated with vehicle and GLP-1.

To investigate whether GIP and GLP-1 modulate sitagliptin-induced resistin levels, we perfused the infarcted hearts with GIP and GLP-1 (Figure 5). The results showed that the level of sitagliptin-induced resistin was decreased by 40% compared with the vehicle group. The effects of GIP on resistin levels were similar to those of sitagliptin. In contrast, GLP-1 administration did not significantly affect the resistin levels. Furthermore, the addition of GIP did not further reduce resistin levels in sitagliptin-treated group.

#### Effect of PI3K/Akt signalling on NGF levels (Experiment 3)

**Figure 6:**
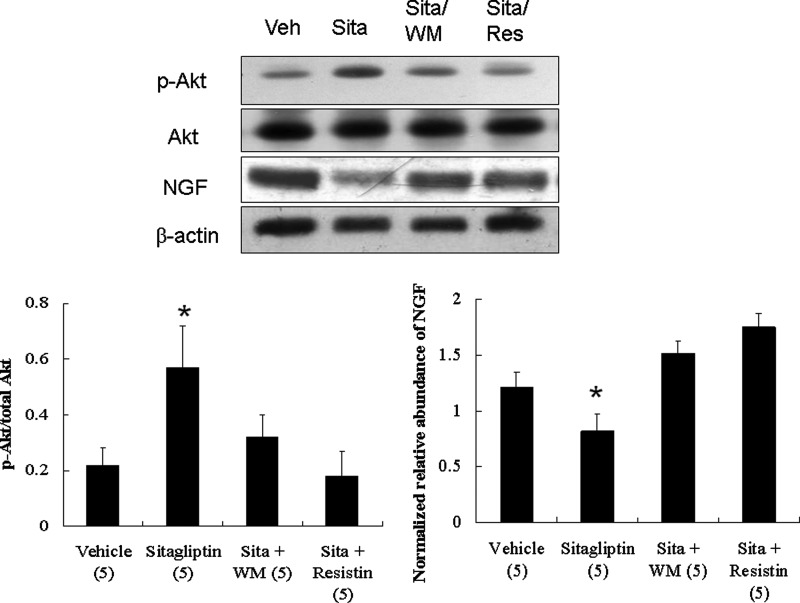
Effect of PI3K/Akt signalling on NGF levels (Experiment 3) In a rat isolated heart model, effect of PI3K signalling and resistin on NGF levels. Compared with sitagliptin-treated infarcted rats alone, significantly increased NGF levels were observed in rats infused with WM (a PI3K antagonist). Resistin significantly increased levels of NGF compared with sitagliptin alone. *P<0.05 compared with the groups treated with vehicle, sitagliptin (sita) + WM, and sitagliptin (sita) + resistin.

To elucidate the role of PI3K/Akt signalling in modulating NGF, we assessed the effects of WM on NGF levels. Western blotting showed that sitagliptin treatment resulted in a significant decrease (*P*<0.01) in NGF level compared with vehicle treatment (Figure 6). This attenuated effect of sitagliptin on NGF level was blocked by WM or resistin infusion, confirming the role of PI3K and resistin in sitagliptin-mediated NGF level.

## DISCUSSION

**Figure 7:**
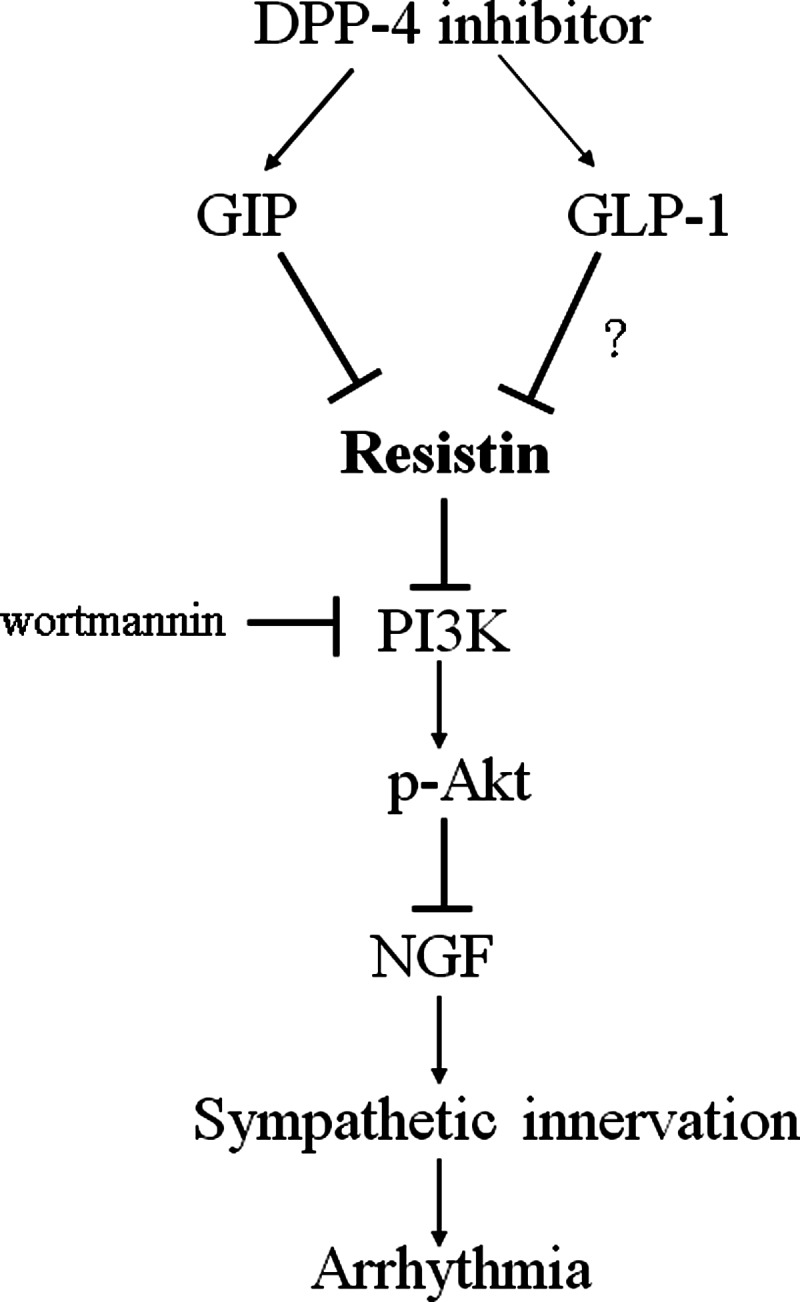
Schematic representation illustrates the involvements of DPP-4 inhibitors and its related components in NGF-related sympathetic innervation in postinfarcted rats Sitagliptin attenuates resistin production through GIP activation. Thereby, resistin inhibits PI3K activation and subsequent Akt phosphorylation, which attenuates NGF expression. Inhibition of these signalling pathways is indicated by the vertical lines.

In the present study, we demonstrated for the first time that infarction was associated with an increased resistin expression via the GIP-dependent pathway, subsequently leading to an increased NGF expression both *in vivo* and *ex vivo*. The non-glycaemic role of sitagliptin and, in particular, its role in the regulation of resistin may allow its participation in the pathogenesis of sympathetic innervation. These results showed the beneficial effects of sitagliptin, as seen structurally by a reduction in cardiac nerve sprouting, molecularly by myocardial resistin and NGF protein and mRNA levels, biochemically by myocardial NE levels, pharmacologically by GIP and GLP-1 infusion, and electrophysiologically by an improvement in fatal ventricular tachyarrhythmias. These findings suggest that sitagliptin has an anti-arrhythmic effect after MI, supporting that sitagliptin may play a role in modifying the high risk of fatal arrhythmias that are inherent in type 2 diabetes [28]. Our results also illustrate that resistin can mediate the effect of sitagliptin on arrhythmias, and the effect of sitagliptin on attenuating sympathetic innervation was supported by three lines of evidence (Figure 7).

1) We found that resistin was expressed in both normal and post-infarcted hearts. The up-regulation and production of resistin represent an intrinsic response against myocardial injury. Our results were consistent with recent findings showing that resistin levels were increased in patients with acute MI [29]. Given the large infarction size in the present study, either sustained cytokine up-regulation or a second wave of cytokine up-regulation corresponded to the chronic remodelling phase [30]. We next examined the functional implications of sitagliptin-attenuated resistin secretion, and found that resistin played an important role in sympathetic innervation and arrhythmias through modulation of NGF levels.

2) Given that sitagliptin administration increased insulin, GLP-1 and GIP levels, one or more of them may be candidates for attenuating the levels of resistin. DPP-4 inhibitors increase the circulating level of GLP-1 and GIP through inhibiting degradation of these incretin hormones. Since activation of the GLP-1 pathway by exenatide can inhibit CCAAT/enhancer-binding protein-α (C/EBP-α) overexpression [31], the beneficial effect of sitagliptin on resistin may also be attributed to activation of the GLP-1 pathway. We found that infusion of infarcted hearts with GIP resulted in similar levels of resistin compared with sitagliptin, whereas GLP-1 did not have such an effect, suggesting that the effects on resistin were GLP-1-independent. GIP may play a role in mediating sitagliptin-attenuated resistin levels. Consistent with previous studies [32], our results showed that the sitagliptin-augmented GIP levels were associated with attenuated resistin levels.

3) We then assessed the role of PI3K/Akt signalling and resistin on NGF levels. Treatment of post-infarcted hearts with resistin (10 nM) resulted in increased levels of NGF compared with sitagliptin alone, confirming that these effects were resistin dependent. Thus, resistin acted as a mediator of sitagliptin-mediated increases in NGF level. In addition, the *ex vivo* experiments showed that blocking PI3K by WM inhibited the attenuated effect of sitagliptin on NGF, indicating that Akt activation is essential to mediate the protective effects of sitagliptin. Our results showed that the infarction-induced NGF increase depended on the PI3K/Akt pathway, and that inhibition of the PI3K pathway increased NGF levels. Sitagliptin compensated for the impairment of PI3K activity and preserved Akt activity.

### Other mechanisms

Our results suggest that the mechanisms of sitagliptin-induced anti-arrhythmias were related to an attenuated NGF expression through a resistin-dependent pathway, and that the resistin expression was coupled to the regulation of GIP. However, we cannot definitively rule out that other potential DPP-4 off-targets exerted an effect on the myocardium such as C/EBP-α inhibition, antioxidation and neuropeptide Y. First, the expression of the resistin gene has been reported to be induced by C/EBP-α [33,34], and DPP-4 inhibitors are known to inhibit the transcriptional activity of C/EBP transcription factors [35]. Thus, C/EBP-α could be another target of sitagliptin in attenuating resistin expression. Second, the DPP-4 inhibitor vildagliptin has been shown to attenuate free radical production during cardiac ischaemia-reperfusion injury in normoglycaemic pigs [36]. We previously demonstrated that antioxidation induced by administering *n*-acetylcysteine can attenuate sympathetic innervation in infarcted rats [37]. Thus, it is possible that sitagliptin may attenuate arrhythmias by enhancing the antioxidation effect. Third, neuropeptide Y, a DPP-4 enzymatic substrate, is an abundant neuropeptide in the central and peripheral nervous system. The protease activity of DPP-4 can be beneficial for the cardiovascular system by cleaving neuropeptide Y. Neuropeptide Y is co-released with NE from sympathetic synapses [38]. Several studies have identified a trophic role for neuropeptide Y in the nervous systems [39], which in turn induces sympathetic hyperinnervation. However, neuropeptide Y levels cannot be a confounding factor of sympathetic innervation because neuropeptide Y level are expected to be increased after adding DPP-4 inhibitors, suggesting that factors other than neuropeptide Y may contribute to the pathogenesis of attenuated sympathetic innervation. Finally, other effects of sitagliptin that may contribute to changes in the effect of NGF on other adipokines that are potentially related to insulin sensitivity such as tumour necrosis factor-α and adiponectin cannot be excluded. Further studies are needed to elucidate the relationship between DPP-4 inhibition, and adipokine and NGF expressions.

### Clinical implication

The present study was undertaken to explore the possibility that sitagliptin may be clinically applicable for anti-arrhythmias after MI. Given the high incidence of coronary artery disease in patients with diabetes mellitus, many factors require clarification before DPP-4 inhibitors can be considered as an adjunctive treatment after MI. The incretin axis (GLP-1, GIP and DPP-4) is widely expressed in the cardiovascular system, and previous studies have focused on the effects of GLP-1. Our results showed that an increase in GIP as a consequence of DPP-4 inhibition may represent an important additional beneficial mechanism. Although whether or not GIP has any biological function in the heart is unknown, the current study on rodents linked GIP activation to resistin and NGF expressions. Our results suggest the pleiotropic effects of DPP-4 inhibition and suggest a potential role for these agents in anti-arrhythmia.

Understanding the underlying molecular mechanisms of the action of resistin may aid in the development of new and effective strategies to control the detrimental effects of resistin on post-infarction remodelling. Therapies targeting reducing resistin may be a promising clinical strategy to deepen the understanding of the role of resistin post-infarction. In addition to new drugs specifically targeting resistin such as antisense oligonucleotides, antibodies and small molecular inhibitors, our results show that DPP-4 inhibition may be another option. Furthermore, if the resistin-induced signalling pathways can be clearly elucidated, additional downstream targets of resistin can be investigated which may lead to the inhibition of resistin with the development of new drugs. A decrease in resistin levels could potentially be beneficial to prevent arrhythmias in pathophysiological disorders such as those experienced post-infarction. In the present study, we assessed the effects of DPP-4 inhibitors on post-infarction sympathetic innervation in rats without diabetes. Based on the encouraging results of the present study, we will continue to assess the effects of these drugs in further diabetic animal studies. Of note, the first clinical trial [SITAgliptin plus GRanulocyte-colony-stimulating factor in patients suffering from Acute Myocardial Infarction (SITAGRAMI)] investigating the effect of a combination of sitagliptin and G-CSF in nondiabetic patients with acute MI has been conducted [40].

### Study limitations

Our results showed that the chronic attenuated expression of resistin was associated with anti-arrhythmias. It is unknown whether these results are applicable to diseased human myocardium because the human homologue of resistin is only 59% identical with mouse resistin at the amino acid level, and because the source of resistin appears to differ between humans and mice [41]. The genes in humans and mice have markedly divergent promoter regions, indicating different mechanisms of regulation, tissue distribution and functions [40]. In addition, there are concerns over whether there is a potential non-physiological effect of resistin produced by large infarctions. However, such analysis can only be performed on tissues from animals that survive. This may have biased the results toward a less severe situation, since only animals that coped well with post-infarction remodelling were investigated. Moreover, the presence of resistin in human mononuclear cells [42] raises the possibility that the presence of myocardial resistin mRNA could have been due to the presence of blood cells. However, previous studies have reported undetectable levels of resistin mRNA in rat blood cells. Thus, the presence of resistin mRNA by RT-PCR in the rat myocardial tissues was not due to contamination by monocytes. Finally, we used a non-diabetic animal model in the present study in order to differentiate potential pleiotropic effects from those solely attributable to improved blood glucose control. We cannot rule out the possibility that the anti-arrhythmic response after MI may vary with diabetes status. All of these factors complicate translating these results to a clinical setting.

### Conclusions

Our results provide new evidence that sitagliptin protects from fatal arrhythmias by attenuating NGF-induced sympathetic innervation via the inhibition of resistin production by GIP. In addition to regulation of post-prandial glycaemia, DPP-4 inhibitors may have pleiotropic effects and may play a role in post-infarction arrhythmias.

## Abbreviations

BW: body weight
DPP-4: dipeptidyl peptidase-4
EBP-α: enhancer-binding protein-α
GIP: glucose-dependent insulinotropic polypeptide
GLP-1: glucagon-like peptide-1
LungW: lung weight
LVEDP: left ventricular end-diastolic pressure
LVESP: left ventricular end-systolic pressure
LVW: left ventricular weight
MI: myocardial infarction
NE: noradrenaline
NGF: nerve growth factor
PI3K: phosphatidylinositol 3-kinase
RVW: right ventricular weight
WM: wortmannin
